# Mental health service users’ responses to anticipated discrimination and the Time to Change program in England

**DOI:** 10.1192/j.eurpsy.2020.114

**Published:** 2020-12-21

**Authors:** Gaia Sampogna, Lucia Gehlen, Vincenzo Giallonardo, Emily J. Robinson, Graham Thornicroft, Claire Henderson

**Affiliations:** 1 Department of Psychiatry, University of Campania “Luigi Vanvitelli,” Naples, Italy; 2 University of Cologne, Cologne, Germany; 3 Biostatistics & Health Informatics Department, Institute of Psychiatry, Psychology, and Neuroscience, King’s College London, London, United Kingdom; 4 Centre for Global Mental Health, Institute of Psychiatry, Psychology, and Neuroscience, King’s College London, London, United Kingdom

**Keywords:** Anti-stigma program, discrimination, mental health service’s users, stigma

## Abstract

**Background:**

Responses to anticipateddiscrimination are common among mental health service users and can have adetrimental impact on their recovery. Since 2009, the Time to Change (TTC)anti-stigma program in England has aimed to improve service users’ empowerment,reducing public stigma and discrimination. In this paper, we aim to evaluatewhether service users’ awareness of TTC is associated with fewer responses toanticipated discrimination.

**Methods:**

We used data collected for the evaluation of TTC from samples of mental health service users interviewed by telephone in annual surveys 2009-2014.

**Results:**

Five thousand and nine hundredand twenty-three participants completed the survey, mainly suffering from mooddisorders (depression, 28.4%, n = 1,681) and schizophrenia related disorders(15.4%, n = 915).

In 23.2% of cases,participants were aware of any aspects of the TTC program, while participationin TTC was reported by 2.6%. Being aware of the TTC program was notsignificantly associated with responses to anticipated discrimination, exceptfor those participating in the TTC campaign in 2013. Stopping oneself fromapplying for work was significantly associated with experienced discriminationin both finding (p &lt; 0.001) and keeping (p &lt; 0.001) a job.Concealing mental health problems was associated with a general experience ofbeing shunned (p &lt; 0.001).

**Conclusions:**

Awareness of a nationalanti-stigma program may not be sufficient to encourage people to seek work/educationor to be open about their illness in situations in which they currentlyanticipate discrimination. There is the need to identify new multi-levelstrategies for challenging anticipated discrimination, even focusing ondifferent target groups.

## Introduction

Stigma is a complex phenomenon resulting by three social-cognitive structures: stereotypes, prejudices, and discrimination [1]. Stigma and discrimination can be an additional burden to people with experience of mental health problems, to the point that many feel like it adversely affects their lives more than the actual symptoms [2,3]. Experiencing stigma or discrimination, both structural and interpersonal, is very common among people with mental health problems [4–6], as is the anticipation of negative responses to one’s mental illness [5,7].

Anticipation of stigma and discrimination has been found to be more common than the actual experience [5,7,8] and can even occur in absence of the latter [7,9]. Anticipated stigma and discrimination have several detrimental consequences on the recovery process [10–13]. Quinn and Chaudoir [14] found that high levels of anticipated stigma predicted heightened psychological distress as well as more self-reported illness symptoms. Moreover, anticipated stigma and discrimination have been found to mediate the relationship between experienced and internalized stigma [15]. The overall consequence of discrimination is the reduction in achieving personal recovery, through the achievement of personal life goals, such as having a satisfying job or a supportive relationship [16–21].

Since 2007, the charities Mind and Rethink Mental Illness have been running Time to Change (TTC) (http://www.time-to-change.org.uk/), the largest ever program in England to reduce mental health stigma and discrimination. TTC aims to reduce stigma and discrimination by raising awareness on mental health, dispelling misbeliefs about mental disorders, promoting positive message of recovery through social media and mass-media, facilitating contact between people with and without mental health problems [1,22–26]. The TTC program consisted of several interventions, including a social marketing campaign, programs for specific target groups (e.g., medical students, trainee teachers and head teachers,employers), local anti-discrimination initiatives, activities to promote social contact and social inclusion of people with severe mental disorders, social contact events organized by a range of stakeholders, and the use of social media such as Twitter and Facebook.

As part of the program evaluation, a series of telephone interviews, called the Viewpoint Survey, was conducted annually between 2008 and 2014, with the primary objective of measuring discrimination experienced by service users [6,24,27]. Consistent with the improvements in stigma-related knowledge, attitudes and intended behavior observed among the general public since 2009, these surveys showed an overall fall in the experience of discrimination between 2008 and 2014 and that fewer respondents stopped themselves from starting a relationship in 2014 compared to 2013 [6]. However, there was no clear pattern of change in terms of this or other responses to anticipated discrimination [28].

Mental health service users aware of TTC reported higher levels of confident behaviors, such as challenging and educating others [27], raising the question of whether awareness of TTC, in terms of having seen social marketing campaign material, or participation in TTC activities may affect responses to anticipated discrimination. If those aware of TTC are more willing to educate or challenge others, they may also be less likely to avoid doing things that are likely to involve disclosure of their mental health problem to others, and less likely to conceal their mental health problem in general; if so this is also likely to apply to those who have taken part in activities run by TTC. Awareness or participation in TTC might also reduce anticipated discrimination if it leads to an expectation that others will be aware of TTC and as a result behave in the positive ways promoted by the campaign, instead of in a discriminatory fashion. Therefore, in this paper we aim to test the following hypotheses:

1. Mental health service users aware of TTC are expected to conceal their mental health problems from others less often, compared to those not aware of the campaign.
2. Mental health service users actively participating in program activities are expected to report fewer levels of anticipated discrimination, in terms of not seeking employment, training or personal relationships.

## Method

Data are taken from the Viewpoint Survey carried out in the period 2009–2014. This timeframe follows the launch of the social marketing campaign, and therefore enable us to assess the level of awareness of the TTC program. Participating mental health service users provided data on experienced discrimination and on responses to anticipated discrimination (i.e., concealment of one’s mental health problem and stopping oneself from starting personal relationships, or pursuing education or occupation), as well as socio-demographic and clinical characteristics.

## Participants

Every year, a different sample was recruited by selecting five National Health Service (NHS) Mental Health trusts (service provider organizations). By covering different geographical regions as well as areas in each quintile of socioeconomic deprivation, we aimed for each sample to be representative of England’s population [29]. The methodology has been reported in detail elsewhere [6,22,24,30].

## Measures

### Sociodemographic and clinical variables

Each year, sociodemographic and clinical characteristics were obtained, including: participant’s diagnosis, agreement with the diagnosis and perceived (dis-)advantage of being given the diagnosis. Also, length of contact with mental health services, experience of involuntary treatment, and current type of mental health care received were obtained. Answers were given based on predefined options, or, in case none of those were applicable, explained individually.

### Experienced and anticipated discrimination

The Discrimination and Stigma Scale-12 (DISC-12) consists of 22 items on negative, mental health-related experiences of discrimination (covering 21 specific life areas, plus one for “other” experience), and four items concerning anticipated discrimination [31]. The items can be grouped in four subscales: Unfair Treatment, Stopping Self, Overcoming Stigma, and Positive Treatment [31].

In the Viewpoint Survey, the “Unfair Treatment” and “Stopping Self” subscales were included to measure experienced and anticipated discrimination, respectively. The subscale “Stopping Self” includes four items for evaluating not doing things in specific areas of life due to the expectation of stigma plus one item on general concealment of one’s mental illness. Responses are given on a four-points scale labeled “not at all,” “a little,” “moderately” and “a lot.” For the "Unfair Treatment" subscale, an additional “not applicable” option can be used where items relate to situations which were not relevant to the participant in the previous 12 months (e.g., in relation to being a parent) or in which a diagnosis could not have been known about.

### TTC program awareness

One item in the Viewpoint survey evaluates the awareness of TTC. Answer options are “No,” meaning that one has not been aware of any aspects of TTC, and alternatively “Yes, I have seen some publicity for the campaign” or “Yes, I have participated in some of the activities.” In case participants indicate participation in program activities, they are asked to specify the type of participation.

## Statistical Analysis

In order to explore socio-demographic characteristics of the sample, appropriate descriptive statistics (chi-square, Fisher’s exact test) were performed. The main outcome measure was the “Stopping Self” subscale of the DISC-12. Due to its psychometric limitations, the four items of the subscale were analyzed separately and managed as dichotomous variables (i.e., “no” response to anticipated discrimination vs. “some” response). Logistic regression models were used, and explanatory variables were added in a forward stepwise method, following Hamilton et al.’s approach [30]. In particular, the following explanatory variables were added: the TTC program awareness (categorical), sociodemographic, and clinical characteristics [30], experienced discrimination (continuous).

Missing values, that is, items left unanswered or refused to answer, were labeled as missing and included in the analyses. An interaction term between study year and TTC program awareness was added to the model. Study year was not included as a predictor *per se*, because the development of anticipated discrimination over time, that is, program years, had already been covered by previous research [6]. Statistical analyses were conducted using IBM SPSS Statistics 20 and Stata version 12.1.

## Results

Between 2009 and 2014, 5,923 participants completed the survey. Response rates ranged from 7% (in 2009) to 11% (in 2011). All socio-demographic details are reported in Table 1. In the majority of the cases, respondents report to suffer from mood disorders (depression, 28.4%, *n* = 1,681; bipolar disorder, 20%, *n* = 1,185), followed by schizophrenia and schizoaffective disorders (15.4%, *n* = 915), anxiety disorders (9.2%, *n* = 547), and personality disorders (7.3%, *n* = 430).

**Table 1.** Socio-demographic characteristics of the sample.

TTC program awareness, *N* (%)		
Not aware of any aspects	4,362	(73.6)
Seen some publicity	1,375	(23.2)
Participated in activities	155	(2.6)
Missing	31	(0.5)
Age, mean (SD)	44.71	(11.25)
Gender, *N* (%)		
Female	3,683	(62.2)
Male	2,224	(37.5)
Transgender	15	(0.3)
Missing	1	(0.0)
Ethnicity, *N* (%)		
White	5,387	(91.0)
Non-white/mixed	499	(8.4)
Missing	37	(0.6)
Employment, *N* (%)		
Unemployed	2,643	(44.6)
Employed	1,511	(25.5)
Volunteering/studying or training/other	1,265	(21.4)
Retired	496	(8.4)
Missing	8	(0.1)
Highest level of education, *N* (%)		
School/O-Levels/equivalent	1,803	(30.4)
College/A-Levels/equivalent	1,573	(26.6)
Undergraduate degree	1,136	(19.2)
Postgraduate degree	665	(11.2)
Professional training	345	(5.8)
Other	353	(6.0)
Missing	48	(0.8)
Active in religion, *N* (%)		
No	3,846	(64.9)
Yes	2,062	(34.8)
Missing	15	(0.3)
Diagnosis, *N* (%)		
Depression	1,681	(28.4)
Bipolar disorder	1,185	(20.0)
Schizophrenia and schizoaffective disorder	915	(15.4)
Anxiety disorder	546	(9.2)
Personality disorder	430	(7.3)
Other	678	(11.4)
Missing	488	(8.2)
Agree with diagnosis, *N* (%)		
Yes	4,861	(82.1)
No	218	(3.7)
Unsure	335	(5.7)
Missing	509	(8.6)
Find diagnosis an advantage, *N* (%)		
Advantage	3,009	(50.8)
No difference	713	(12.0)
Disadvantage	1,081	(18.3)
Missing	1,120	(18.9)
Years since first contact with MH services, mean (SD)	14.06	(11.05)
Missing	541	
Previous involuntary treatment, *N* (%)		
No	3,773	(63.7)
Yes	2,139	(36.1)
Missing	11	(0.2)
Current main type of MH care, *N* (%)		
Outpatient/ambulatory	4,877	(82.3)
Treatment at home	584	(9.9)
Day care	113	(1.9)
Other	333	(5.6)
Missing	16	(0.3)
DISC score, mean (SD)	31.0	(22.92)

Abbreviation: MH, mental health.

Most respondents (73.6%, *n* = 4,362) were not aware of any aspects of the TTC program. Exposure to publicity for the campaign was reported by 23.2% (*n* = 1,375). Participation in program activities was reported by only 2.6% (*n* = 155) (Table 1).

Most participants reported to generally conceal or hide one’s mental health problem (74.3%, *N* = 4,316), stopping themselves from applying for work (61.9%, *n* = 2,666) and from having a closer personal relationship (52.6%, *n* = 2,803).

## Relationship Between TTC Campaign Awareness/Participation and Levels of Anticipated Discrimination

The majority of mental health service users actively participating in program activities did not reported anticipated discrimination in training and educational activities (vs. 45.6% in people not aware of the program, *p* < 0.000). On the other hand, those participating in the TTC activity reported in 52.9% of cases experiences of anticipated discrimination related to working (vs. 43.2% reported by users not aware of any aspects of the campaign, *p* < 0.000) and in personal relationships (52.3 vs. 46.0% in those not aware of any aspects, *p* < 0.000) (Table 2).

**Table 2.** Differences in anticipated discrimination according to the levels of TTC program awareness.

TTC program awareness		Stop working		Stop educating		Stop relationship		Concealment	
		*N*	%	*N*	%	*N*	%	*N*	%
Not aware of any aspects	Any anticipated discrimination	1,885	43.2	1,315	30.1	2,007	46.0	3,124	71.6
	No anticipated discrimination	**1,231**	**28.2**	**1,991**	**45.6**	**1,905**	**43.7**	**1,152**	**26.4**
	Missing	1,246	28.6	1,056	24.2	450	10.3	86	2.0
	Total	4,362	100.0	4,362	100.0	4,362	100.0	4,362	100.0
Seen some publicity	Any anticipated discrimination	691	50.3	448	32.6	703	51.1	1,062	77.2
	No anticipated discrimination	**369**	**26.8**	**665**	**48.4**	**552**	**40.1**	**293**	**21.3**
	Missing	315	22.9	262	19.1	120	8.7	20	1.5
	Total	1,375	100.0	1,375	100.0	1,375	100.0	1,375	100.0
Participated in activities	Any anticipated discrimination	82	52.9	48	31.0	81	52.3	114	73.5
	No anticipated discrimination	**32**	**20.6**	**89**	**57.4**	**60**	**38.7**	**41**	**26.5**
	Missing	41	26.5	18	11.6	14	9.0	155	100.0
	Total	155	100.0	155	100.0	155	100.0	16	50.0
Missing	Any anticipated discrimination	8	25.0	7	21.9	12	37.5	11	34.4
	No anticipated discrimination	11	34.4	15	46.9	13	40.6	5	15.6
	Missing	13	40.6	10	31.3	7	21.9	32	100.0
	Total	32	100.0	32	100.0	32	100.0	3,124	71.6
	**	χ2(6) = 35.018 p &lt; 0.000		*χ* ^2^(6) = 30.334, *p* < 0.000		*χ* ^2^(6) = 18.256, *p* < 0.006		*χ* ^2^(6) = 55.469, *p* < 0.000	

*p* <0.000=.

Unemployed/retired service users with a diagnosis of depression, bipolar disorder, or personality disorder were most likely to stopping oneself applying for work (Table 3).

**Table 3.** Univariate analyses.

Variable	Applying for work		Applying for education or training courses		Having a close personal relationship		Concealing/hiding one’s mental health problem	
	RAD (%)	Association	RAD (%)	Association	RAD (%)	Association	RAD (%)	Association
Age		*χ* ^2^(47) = 90.72 *p* < 0.001 *η* = 0.151		*χ* ^2^(47) = 63.29 *p* = 0.057		*χ* ^2^(47) = 52.37 *p* = 0.274		*χ* ^2^(47) = 112.30 *p* < 0.001 *η* = 0.145
				
Gender								
Female	62.6	*p* = 0.250, Fisher’s exact test	40.8	*p* = 0.179, Fisher’s exact test	53.1	*χ* ^2^(2) = 1.33 *p* = 0.515	78.3	*p* < 0.001, Fisher’s exact test
Male	60.7		37.9		51.6		67.6	
Transgender	42.9		40.0		45.5		71.4	
Ethnicity								
White	61.9	*χ* ^2^(1) = 0.13 *p* = 0.718	39.5	*χ* ^2^(1) = 0.58 *p* = 0.448	52.4	*χ* ^2^(1) = 1.29 *p* = 0.256	74.6	*χ* ^2^(1) = 3.76 *p* = 0.052
Non-white/mixed	61.0		41.4		55.2		70.6	
Employment								
Unemployed	69.0	*χ* ^2^(3) = 126.37 *p* < 0.001	48.2	*χ* ^2^(3) = 144.74 *p* < 0.001	58.1	*χ* ^2^(3) = 114.78 *p* < 0.001	74.1	*χ* ^2^(3) = 29.10 *p* < 0.001
Employed	49.7		27.1		42.7		78.7	
Volunteering/studying or training/other	64.6		37.5		57.2		71.7	
Retired	54.1		40.6		41.4		68.0	
Highest level of education								
O-Levels/ Equivalent	64.0	*χ* ^2^(5) = 4.06 *p* = 0.540	45.6	*χ* ^2^(5) = 66.56 *p* < 0.001	52.9	*χ* ^2^(5) = 8.84 *p* = 0.116	70.5	*χ* ^2^(5) = 26.95 *p* < 0.001
A-Levels/ Equivalent	62.0		41.5		53.3		75.2	
Undergraduate degree	60.0		31.6		51.8		77.6	
Postgraduate degree	60.6		32.8		48.1		77.8	
Professional training	60.9		36.2		51.8		75.4	
Other	61.1		48.2		57.5		71.4	
Active in religion								
No	62.7	*χ* ^2^(1) = 2.27 *p* = 0.132	40.3	*χ* ^2^(1) = 1.06 *p* = 0.303	53.7	*χ* ^2^(1) = 4.90 *p* = 0.027	75.6	*χ* ^2^(1) = 9.41 *p* = 0.002
Yes	60.4		38.7		50.5		71.9	
Diagnosis								
Depression	60.5	*χ* ^2^(5) = 25.87 *p* < 0.001	39.8	*χ* ^2^(5) = 37.51 *p* < 0.001	53.1	*χ* ^2^(5) = 60.47 *p* < 0.001	77.5	*χ* ^2^(5) = 71.24 *p* < 0.001
Bipolar disorder	63.2		36.3		45.4		69.6	
Schizophrenia and schizoaffective disorder	60.4		38.4		53.2		66.8	
Anxiety disorder	64.5		43.8		53.9		79.1	
Personality disorder	73.8		53.5		67.8		83.0	
Other	57.8		36.4		53.3		73.4	
Agree with diagnosis								
Yes	62.0	*χ* ^2^(2) = 1.76 *p* = 0.415	39.2	*χ* ^2^(2) = 9.88 *p* = 0.007	52.2	*χ* ^2^(2) = 5.56 *p* = 0.062	74.3	*χ* ^2^(2) = 2.93 *p* = 0.231
No	67.1		45.4		59.9		69.3	
Unsure	63.3		47.8		55.9		75.5	
Find diagnosis an advantage								
Advantage	60.5	*χ* ^2^(2) = 19.36 *p* < 0.001	37.9	*χ* ^2^(2) = 16.63 *p* < 0.001	49.2	*χ* ^2^(2) = 33.67 *p* < 0.001	72.7	*χ* ^2^(2) = 7.67 *p* = 0.022
No difference	68.4		45.5		60.1		77.0	
Disadvantage	58.3		36.8		51.1		72.8	
Years since first contact with MH services		*p* = 0.001, Fisher’s exact test *η* = 0.149		*p* = 0.300, Fisher’s exact test		*p* = 0.134, Fisher’s exact test		*p* = 0.149, Fisher’s exact test
				
Previous involuntary treatment								
No	61.2	*χ* ^2^(1) = 1.52 *p* = 0.217	39.1	*χ* ^2^(1) = 1.13 *p* = 0.287	52.5	*χ* ^2^(1) = 0.02 *p* = 0.893	77.2	*χ* ^2^(1) = 47.24 *p* < 0.001
Yes	63.1		40.7		52.7		69.0	
Current main type of MH care								
Outpatient/ambulatory	62.4	*χ* ^2^(3) = 18.68 *p* < 0.001	40.1	*χ* ^2^(3) = 14.19 *p* = 0.003	52.6	*χ* ^2^(3) = 6.24 *p* = 0.101	75.0	*χ* ^2^(3) = 15.26 *p* = 0.002
Treatment at home	64.9		43.6		54.5		73.1	
Day Care	62.5		31.0		57.8		60.2	
Other	48.9		30.6		46.5		70.9	
DISC Score		*p* < 0.001, Fisher’s exact test *η* = 0.348		*p* < 0.001, Fisher’s exact test *η* = 0.361		*p* < 0.001, Fisher’s exact test *η* = 0.385		*p* < 0.001, Fisher’s exact test *η* = 0.272
				

Abbreviations: MH, mental health; RAD response to anticipated discrimination; TTC PA, Time to Change Program Awareness.

## Impacts of Levels of TTC Campaign Awareness on Probability to Conceal

Service users who participated in program activities in 2013 were 46% less likely to conceal their mental health problems compared to service users with any other form of TTC program awareness in other study years (Confidence Interval, CI: 32.7–65.6%, *p* < 0.05). For the other years of the study considered, no significant associations between campaign awareness and concealment were found (Table 4).

**Table 4.** Logistic regression model.

Variable	*B*	Concealment
Gender, ref. category: female		
Male	−0.425	0.654 (0.575–0.743)*
Transgender	−0.472	0.624 (0.184–2.111)
Active in religion (ref. category: No)		
Yes	−0.145	0.865 (0.761–0.984)*
Missing	−1.096	0.334 (0.106–1.058)
Employment (ref. category: Unemployed)		
Employed	0.362	1.436 (1.218–1.693)*
Volunteering/studying or training/other	−0.043	0.958 (0.817–1.124)
Retired	−0.148	0.862 (0.690–1.078)
Missing	0.273	1.314 (0.263–6.567)
Diagnosis		
Bipolar disorder	−.0329	0.720 (0.599–0.864)*
Schizophrenia and schizoaffective disorder	−0.219	0.804(0.655–0.985)*
Anxiety disorder	0.035	1.035 (0.810–1.324)
Personality disorder	0.145	1.157 (0.862–1.551)
Other	−0.202	0.817 (0.658–1.014)
Missing	0.015	1.015 (0.791–1.303)
Previous involuntary treatment (ref. category: No)		
Yes	−0.357	0.700 (0.610–0.803)*
Missing	−0.955	0.385 (0.095–1.557)
Current main type of MH care (ref. category = Outpatient/ambulatory*)*		
Treatment at home	−.0.023	0.977 (0.793–1.204)
Day care	−0.530	0.589 (0.393–0.882)*
Other	−0.256	0.774 (0.597–1.005)
Missing	−0.683	0.505 (0.171–1.489)
DISC Score	0.024	1.024 (1.021–1.027)*
Awareness TCC*year of the campaign (base = not aware of any aspects* 2009)		
Seen publicity*2010	0.220	1.246 (0.857–1.812)
Seen publicity*2011	0.197	1.218 (0.816–1.818)
Seen publicity*2012	0.302	1.353 (0.970–1.888)
Seen publicity*2013	−0.003	0.997 (0.732–1.357)
Seen publicity*2014	0.173	1.189 (0.862–1.640)
Participated in activities*2010	0.466	1.593 (0.518–4.900)
Participated in activities*2011	−0.271	0.763 (0.285–2.044)
Participated in activities*2012	1.354	3.873 (0.488–30.744)
Participated in activities*2013	−0.769	0.463 (0.234–0.917)*
Participated in activities*2014	−0.252	0.777 (0.353–1.713)
Missing*2010		0.000
Missing*2011	−0.020	0.980 (0.071–13.565)
Missing*2012	−1.455	0.233 (0.033–1.675)
Missing*2013	−1.232	0.292 (0.054–1.572)
Missing*2014	−2.410	0.090 (0.008–1.055)

* *p* < 0.005.

Moreover, regarding the concealment of mental disorder, service users who were male (CI: 25.7–42.5%, *p* < 0.001), affected by bipolar disorder (CI: 1.5–34.5%, *p* < 0.05), with previous involuntary admission (CI: 19.7–39%, *p* < 0.001) and active in religion (CI: 6.0–23.9%, *p* < 0.05) have a decreased probability of concealing their mental disorder (Table 3).

## Other Factors Related to Responses to Anticipated Discrimination

Stopping oneself from applying for work was significantly associated with experienced discrimination in both finding (*p* < 0.001) and keeping (*p* < 0.001) a job. Concealing one’s mental health problems was significantly associated with a general experience of being shunned (*p* < 0.001). Stopping oneself from applying for education and training was significantly associated with experienced discrimination in education (*p* < 0.001). Stopping oneself from having intimate personal relationships was significantly associated with experienced discrimination in making and keeping friends (*p* < 0.001) as well as dating (*p* < 0.001).

## Discussion

The present study is the first aiming to investigate responses to anticipated discrimination in a large sample of mental health service users participating to an anti-stigma program. In this study, being aware of the TTC program was found not to be a predictor of responses to anticipated discrimination. Only in 2013, a small percentage of participants reported active participation in the program activities, which was significantly associated with a reduced likelihood of concealing one’s mental health problem. It could be that such an effect is related to the different messages launched by the campaign over time; considering that in 2013 a video on the story of a mental health users affected by schizophrenia was shared on-line. This finding deserves more investigation since it could be that a specific activity released in that year could have reached more people.

Moreover, being aware of the TTC program was not associated with a reduction of stopping oneself from the pursuit of educational, occupational, or personal relationship opportunities. This finding must be considered carefully, since many factors may have an influence on the development of these responses, as explained by the model of self-esteem and self-stigmatization. In particular, Abiri et al. [32] reported that people with greater insight of the mental health stigma could find it more difficult to limit their impact. It could be that anti-stigma programs are useful tool for improving positive coping strategies such as challenging and educating others [27], whereas the reduction of the levels of self-stigma might require more specialized psychosocial interventions [32–38].

In terms of socio-demographic predictors of responses to anticipated discrimination, this study found a higher likelihood of concealment in service users who were female and employed, confirming data by Farrelly et al. [5]. We found that service users active in religion had a slightly lower likelihood to conceal their mental health problems. We know that religiosity and religious coping can have a positive impact on long term well-being of people with mental health problems and their family members [36]. This association between religiosity, as well as other related areas such as spirituality, and concealment has never been investigated previously, but this should be a useful area for future research.

Low level of concealment was reported by service users who had previously experienced involuntary treatment or access day care facilities. This finding is not in line with Rüsch et al. [37], who found that disclosure appears to be harder for individuals with recent psychiatric inpatient treatment. Furthermore, this finding should be carefully explored in order to clarify the impact of involuntary treatments, levels of perceived coercion, style of clinical decision-making on the levels of anticipated discrimination in patients with severe mental disorders [38–42].

Educational and occupational level were significantly associated with stopping oneself from pursuing educational/working opportunities, this may be due to previous negative discriminating experiences [43–45]. It has been extensively reported that having an occupation creates a sense of confidence in people that empowers them to further pursue their educational development [46].

Finally, people suffering from affective disorders or personality disorders reported higher levels of anticipated discrimination related to educational activities and in stopping themselves in searching for these activities as found by Rossi et al. [46,47].

## Strengths and Limitations

This study examined responses to anticipated discrimination in a sample that was much larger than samples used in previous research on the topic (e.g., [5,7,48]). However, a selection bias could have affected our study and limit the generalizability of findings. In particular, it should be noted that mental health service users experiencing or anticipating higher levels of discrimination were more prone to take part in the survey and are thus overrepresented, which would again limit the generalizability of the present findings [49].

Finally, due to the psychometric properties of the “Stopping Self” subscale, the use of an overall score was not recommended [31] and items were analyzed separately. This methodological choice limited options for precise statistical analyses as variance observed in the data could only be small, particularly with the short 4-point scale used. It must be considered that the use of single item measurement scales is not necessarily inferior to multiple item measurement [50,51]. It may be useful to develop a new assessment tool tailored on the evaluation of responses to anticipated discrimination, satisfying the common psychometric requirements [52].

## Implications and Further Research

Based on our findings, being aware of the TTC anti-stigma program had no effect on the pursuit of life opportunities for mental health services users, and therefore there is the need to identify new strategies for challenging anticipated discrimination. In particular, it is necessary to develop interventions targeted to the reduction of anticipated mental health discrimination, for example, health professionals could be trained to address the anticipation of stigma and discrimination [53–59]. Moreover, these findings suggest further development of anti-stigma programs in order to promote a heightened sense of empowerment in service users, for example, encouraging the pursuing of specific areas in life.

Due to their high prevalence and adverse consequences, it is time that anticipated discrimination and related concepts such as self-stigma gains much-needed attention. This may be achieved if recommendations from ROAMER Consortium are followed as research in stigma and discrimination represent one of the most urgent priorities for research in psychiatry in the next years [60–63].

## Data Availability

The DISC measure is available subject to terms and conditions at http://www.kcl.ac.uk/ioppn/depts/hspr/research/ciemh/cmh/CMH-Measures.aspx. The Viewpoint dataset is not available. Data sharing would first require an application to the UK’s Health Research Authority, since the research team does not have the capacity to do now it considering that the Viewpoint survey has been discontinued.
